# Mathematical Modeling of the Effect of Pulsed Electric Field on the Specific Permselectivity of Ion-Exchange Membranes

**DOI:** 10.3390/membranes11020115

**Published:** 2021-02-06

**Authors:** Andrey Gorobchenko, Semyon Mareev, Victor Nikonenko

**Affiliations:** Membrane Institute, Kuban State University, 149 Stavropolskaya St., 350040 Krasnodar, Russia; gorobchenkoandrey@mail.ru (A.G.); mareev-semyon@bk.ru (S.M.)

**Keywords:** electrodialysis, ion-exchange membrane, pulsed electric field, ion transfer, numerical simulation

## Abstract

The application of pulsed electric field (PEF) in electrodialysis has been proven to be efficient for a number of effects: increasing mass transfer rate, mitigation of scaling and fouling, reducing water splitting. Recently, the improvement of the membrane permselectivity for specific counterions was discovered experimentally by the group of Laurent Bazinet (N. Lemay et al. *J. Memb. Sci.* 604, 117878 (2020)). To better understanding the effect of PEF in electrodialysis, simulations were performed using a non-stationary mathematical model based on the Nernst–Planck and Poisson equations. For the first time, it was not only the condition used when the current density is specified but also the condition when the voltage is set. A membrane and two adjacent diffusion layers are considered. It is shown that when applying the regime used by Lemay et al. (the same current density in conventional continuous current (CC) mode and during the pulses in PEF mode), there is a significant gain in specific permselectivity. It is explained by a reduction in the membrane concentration polarization in PEF mode. In the CC mode of electrodialysis, increasing current density leads to a loss in specific permselectivity: concentration profiles in the diffusion layers and membrane are formed in such a way that ion diffusion reduces the migration flux of the preferentially transferred ion and increases that of the poorly transferred ion. In PEF mode, the concentration profiles are partially restored during the pauses when the current is zero. However, if a different condition is used than the condition applied by Lemay et al., that is, when the same average current density is applied in both the PEF and CC modes, there is no gain in specific permeability. It is shown that within the framework of the applied mathematical model, the specific selectivity depends only on the average current density and does not depend on the mode of its application (CC or PEF mode).

## 1. Introduction

Electrodialysis (ED) is one of the rapidly progressing membrane methods for desalination, concentration and separation of aqueous solutions today. Many years of application of this method in various industries has proven its efficiency [1,2,3,4]. Although ED is a mature method, it has some hindrances. Most of them are associated with the phenomenon of concentration polarization, which is caused by the difference in the transport numbers of ions in the solution and the ion-exchange membrane. The effect leads to a decrease in the electrolyte concentration in a thin layer near the membrane surface. The growth of the system resistance results in the current density decrease with time in the potentiostatic mode or an increase in the potential drop with time in the galvanostatic mode [5,6]. Depending on the mode, the system tends to a certain stationary value of the current density or potential drop. This value can be considered as a maximum of ED productivity under given conditions (constant current or potential drop). The concentration polarization provides such undesirable effects as water splitting [7,8,9,10], membrane scaling [7,11,12] and fouling [13].

The use of current or voltage pulses alternating with pauses in ED, the so-called PEF mode, leads to the mitigation of concentration polarization and, as a result, improves the performance of ED compared with conventional CC mode [5,7,8,13,14,15]. In particular, it is found that the use of PEF mode allows increasing mass transfer rate [16,17], can essentially reduce water splitting [8,17], membrane scaling [7,18] and fouling [13,19,20], as well as membrane stack resistance [21].

Mishchuk et al. [5] showed theoretically that the characteristic pulse time should be less than the transition time required to build up the concentration polarization layer near the membrane. The pause duration should be comparable to the pulse duration to enable the concentration profiles that can be restored to their initial state. However, the pause should not be too long; otherwise, diffusion and osmotic fluxes in the membrane counteract the desalination process. The gain in mass transfer in PEF mode mainly occurs at the beginning of the pulse lapse due to the appearance of a current spike (or voltage fall) for a short period of time. Based on a mathematical description using the Nernst–Planck equations, Sistat et al. [16] explain that this current spike (or voltage fall) is due to the partial concentration restoration at the vicinity of the membrane during the pause lapse. Moya and Moleón [22] applied the network simulation method for examining the time variation of the counterion flux leaving the membrane in response to a pulsed electric potential as well as some other parameters in order to establish the conditions under which the application of PEF can improve the performance of electrodialysis.

Uzdenova et al. [23] theoretically studied the effect of a PEF on mass transfer at overlimiting current regimes by applying the Nernst–Planck–Poisson–Navier–Stokes equations. They showed that the intensive electroconvective vortices decay almost immediately after switching off the current. However, the residual weak vortices continue to exist near the membrane surface for a few tenths of a second, fed by a non-uniform concentration field. After the pause, when the voltage pulse resumes, the first weak vortices appear already in 0.01 s. This rapid recovery of electroconvection is explained by the inhomogeneity of the residual concentration field. The inhomogeneity stimulates the development of electroconvection in the same way as it occurs in the case of an electrically heterogeneous surface [24]. Later, similar effects were obtained experimentally and described by Butylskii et al. [25]. Zyryanova et al. [26] showed that in overlimiting PEF modes, the average current density increases up to 33% over the period when applying the same average voltage in PEF and CC modes. Lemay et al. [27] showed that the use of PEF mode allows reducing energy consumption for the demineralization process. Thus, these results showed that the application of the PEF modes in ED is a promising way for the process intensification in the overlimiting current range. It is hypothesized [26,27] that the main cause of improvement of ED performance when applying PEF in intensive current regimes is electroconvection.

Currently, the main effect of PEF on the process of ED treatment of solutions is the mitigation or suppression of the membrane scaling and fouling. This effect was well established experimentally by a number of authors [7,11,13,18,19,20,28,29,30]. The explanation of the impact of PEF is as follows:

- Reduction of concentration polarization. Relaxation of the concentration profile occurs at the membrane surface during the pause lapse. The concentrations of ion species resume partially or completely to the initial values;
- Intensification of electroconvection at overlimiting current regimes, which in addition to the increase in mass transfer helps to wash out the scale and foulant components from the membrane surface;
- Reduction of water splitting (pH values at which some components can precipitate are not reached).

Recently, Lemay et al. [17] experimentally established another important positive effect of PEF mode on ED characteristics. It was found that the use of PEF mode allows increasing the specific permselectivity of ion-exchange membranes (preferential transport of counterions of one kind over counterions of another kind). In CC mode, a high specific permselectivity (sometimes allowing the flux of one kind of ions to be >100 times higher than the flux of another one at the equal bulk concentrations [31,32]) can be obtained only at low current densities, *i*. When *i* approaches its limiting value, *i*_lim_, the specific selectivity is lost [33,34] due to increasing concentration polarization and the transition of the ion transfer control from the membrane to the depleted diffusion layer [35]. The possibility to have a high permselectivity at elevated current densities is of great practical interest. The authors associated the enhancement of permselectivity by PEF with the partial restoration of ion concentration profiles in the depleted boundary solution during the pause. As a consequence, the membrane partially restores control over the kinetics of ion separation. Earlier [17], was studied only the mode where a constant electric current was applied during the pulses. The concentration profiles were calculated, and it was found that during one pulse, there is a gain in the membrane specific permselectivity compared to the conventional continuous current ED mode. However, the averaged in time-specific permselectivity was not quantified. In this paper, a simulation was carried out using a non-stationary mathematical model based on the Nernst–Planck and Poisson equations. For the first time, two PEF modes are compared when current or voltage pulses are applied.

## 2. Experimental Results

Here are some of the experimental results of Lemay et al. [17] showing enhancement of the specific permselectivity by applying PEF in ED of sweet whey. The feed solution was obtained by dissolution of sweet whey powder in distilled water; the total solids content of the obtained solution was 6.5%. The kinetics of desalination of this solution was studied [17] in a batch ED process using a constant current density of 8.0 mA/cm^2^. According to the estimation by the method of Cowan and Brown, the limiting current density was 13.5 mA/cm^2^; hence the ratio of the current density to its limiting value at the beginning of the desalination process was *i/i*_lim_ ≈ 0.6. However, as far as the solution was desalted, the value of *i*_lim_ decreased. When the total degree of desalination was 60%, *i* approached *i*_lim_, and *i/i*_lim_ became approximately equal to 1.5. The same current density of 8.0 mA/cm^2^ was applied during the pulses in PEF mode, while the current was zero during the pauses.

Table 1 compares the demineralization rates found for different ions constituting the sweet whey at two different total degrees of desalination (DD). The ratios of DD by individual ions to the total DD of sweet whey, *θ_k_*, are given in CC mode and in PEF mode for the pulse/pause combination, 1 s–1 s (0.5 Hz).

The greater the *θ_k_* value, the greater the transport number of ion *k* (the fraction of the electric charge transported by this ion through the membrane).

The K^+^ ion is the dominant cation in the sweet whey. Since its mobility in the membrane is relatively high, the demineralization rate for this ion is the highest. The DD by this ion is higher than the total DD, *θ_K_* > 1, and this parameter changes a little with decreasing the total concentration and increasing the *i/i*_lim_ ratio. In CC mode, the values of *θ_Na_* and *θ_Ca_* are close at *i/i*_lim_ ≈ 0.6, but *θ_Na_* is significantly higher than *θ_Ca_* at *i/i*_lim_ ≈ 1.5. That is, with an increase in the *i/i*_lim_ ratio, the selectivity of Ca^2+^ transfer through the membrane decreases in favor of Na^+^. A similar situation occurs when comparing the competitive transport of Na^+^ and Mg^2+^. In the study by Lemay et al., a cation-exchange membrane was used whose properties were close to that of the Neosepta CMX membrane [17]. It is a homogenous membrane (produced by Astom, Tokuyama Soda, Japan) made by the “paste method” [36,37]. Initially, the paste contains styrene monomer with functional groups (which are subsequently grafted with ion-exchange groups), divinylbenzene (45–65%) as a crosslinking agent, a radical polymerization initiator and powdered polyvinyl chloride (45–55%). The paste is deposited on the reinforcing polyvinyl chloride fabric. The copolymerization is carried out before the sulfonation. The membrane consists of two interpenetrating phases: ion exchange material and polyvinyl chloride, PVC, having a particle diameter of 100 nm or less [38]. The structural inhomogeneities in the membrane volume do not exceed 1 micron [39]. An exception is a reinforcing fabric having fibers of 25–30 µm in diameter [40]. The results presented in Table 1 show that in CC mode, this permselectivity decreases both in the cases of Na^+^ and Ca^2+^ and Na^+^ and Mg^2+^. However, in PEF mode, the membrane permselectivity for the Ca^2+^ and Mg^2+^ cations is significantly greater than that in CC mode if the same current density is applied in CC mode and in PEF mode during the pulses. Note that the average current density applied in PEF mode was two times lower since, during the pauses, the current was zero. It is important that the total duration of the ED process needed to obtain total DD ≈ 70% is nearly the same in both modes [17], whereas in PEF mode, the current flows across the membranes only for a certain fraction of the total processing time.

## 3. Theoretical Part

**Figure 1:**
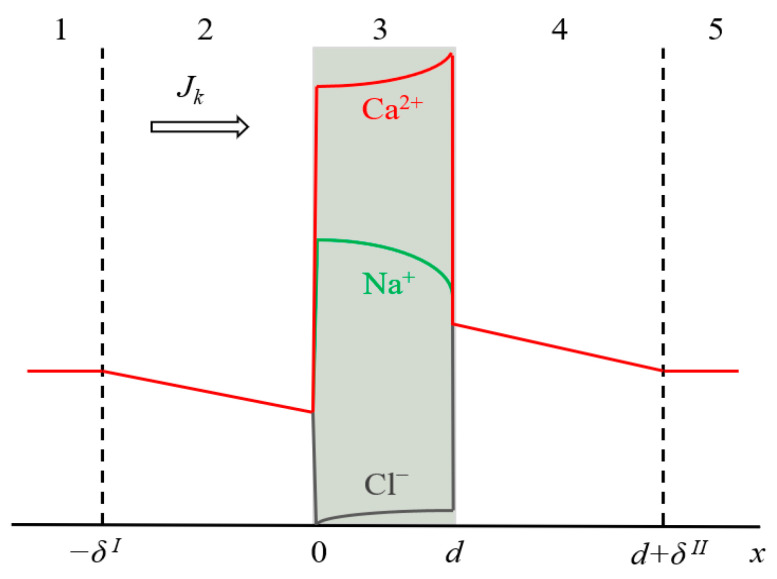
Schematic representation of the simulated system, including an ion-exchange membrane of thickness *d* (3) and two DBLs (2 and 4) of the thickness *δ^I^* and *δ^II^*, respectively. Zones (1) and (5) are the bulk solution. The typical concentration profiles are given in the case of CC mode: for ion *k* in the diffusion layers (k = Na^+^, Ca^2+^ or Cl^−^), and for all three ions in the membrane.

The system under study has a three-layer geometry (an ion-exchange membrane and two adjacent diffusion boundary layers, DBLs) (Figure 1). The membrane is considered as a homogeneous medium, in which fixed charged groups are uniformly distributed. It is assumed that the solvent flux through the membrane is negligible. The impact of convection transport in solution is taken into account implicitly through the diffusion layer thickness, which is considered independent of the current/voltage applied. Water splitting and electroconvection are not taken into account.

Ion transfer in the system is described by the Nernst–Planck and Poisson equation, as well as the material balance equation (the continuity equation of matter conservation):

(1) $$J_{k}^{}=-D_{k}^{}\left(\left(1+\frac{\partial \ln\gamma_{k}}{\partial \ln c_{k}}\right)\frac{\partial c_{k}^{}}{\partial x}+z_{k}c_{k}^{}\frac{\partial \varphi }{\partial x}\right),$$

(2) $$\varepsilon \varepsilon_{0}\frac{\partial E}{\partial x}=F\left(\sum_{k=1}^{3}z_{k}c_{k}^{}+z_{m}\bar{Q}\right),$$

(3) $$\frac{\partial c_{k}}{\partial t}=-divJ_{k},$$

where *J_k_*, *c_k_*, *D_k_*, *z_k_*, and *γ_k_* are the flux density, concentration, diffusion coefficient, charge number and activity coefficient of ion *k* (*k* = Na^+^, Ca^2+^, ${\mathrm{Cl}}^{-}$), respectively; *R* is the gas constant; *T* is the temperature; *F* is the Faraday constant; *φ* is the electric potential; *ε*_0_ is the vacuum permittivity; *ε* is the solution relative permittivity; $E=-\frac{\partial \varphi }{\partial x}$ is the electric field strength; *z_m_* is the charge number of the membrane; $\bar{Q}$ is the exchange capacity of the membrane; *F* is the Faraday constant; *t* is the time.

The electric current density, *i*, in the system is equal to the sum of ionic fluxes (expressed in appropriate units):

(4) $$i=F\sum_{k}z_{k}J_{k},$$

Equation (4) does not take into account the displacement electric current. In the present work, the processes with a characteristic time of more than 10^−2^ s are considered, while the displacement current occurs in a very short time interval, less than 10^−4^ s. This process should be accounted for when considering, for example, a high-frequency impedance of membrane systems [41].

Let *δ^I^* and *δ^II^* be the thicknesses of the depleted and enriched diffusion layers, respectively, and the origin of coordinates is at the solution/membrane boundary (Figure 1). Then, the boundary conditions for the system under study will be:

(5) $$c_{k}\left(x=-\delta^{I}\right)=c_{k}\left(x=d+\delta^{II}\right)=c_{k0},$$

(6) $$\varphi \left(x=-\delta^{I}\right)=0,$$

where $c_{k0}$ is the concentration of ion *k* in the bulk solution.

Equation (5) sets the concentration of all ions in the electroneutral bulk solutions on both sides of the membrane. At the membrane/solution interfaces, the continuity of concentrations and electric potential are assumed, i.e., the functions *c_k_* (*x*, *t*) and *φ* (*x*, *t*) are continuous on the interval (*x* = −*δ ^I^*, *x* = *d* + *δ^II^*). The potential is assumed zero at the left edge of the three-layer system (Equation (6)).

Earlier, we pointed out that our study uses two PEF modes when current or voltage pulses are applied. Equations (1)–(6) are common for both cases.

However, when current pulses are applied, the following boundary condition is used at the right-hand edge of the three-layer system:

(7) $${\left(\frac{\partial \varphi }{\partial x}\right)}_{x=d+\delta^{II}}=-\frac{\frac{RT}{F}\left(\frac{i}{F}+\sum_{k=1}^{3}z_{k}D_{k}\frac{\partial c_{k}}{\partial x}\right)}{\sum_{k=1}^{3}z_{k}^{2}D_{k}c_{k}},$$

where the electric current density, *i*, is set as a known function of time; it takes a given value during a pulse lapse and is zero during a pause lapse.

Equation (7) follows from the continuity of current density and Equations (1) and (4), which were proposed by Uzdenova et al. [42].

In the PEF mode, when voltage pulses are applied, the following boundary condition is used at the right-hand edge of the three-layer system during the pulse:

(8) $$\varphi \left(x=d+\delta^{II}\right)=U_{pulse}=U_{\mathrm{av}}/\alpha ,$$

where *U_pulse_* and *U*_av_ are the voltage applied during the pulse, and the average voltage, respectively; α is the duty cycle.

During the pause, the current density is zero and condition (7) is used, in which *i* is set to zero, *i* = 0.

Thus, the mathematical formulation of the problem for PEF mode, when current pulses are applied, is described by Equations (1)–(7); for the case where voltage pulses are applied, it is described by Equations (1)–(6), (8) during the pulse lapse and Equations (1)–(7) during the pause lapse. The numerical solution was obtained using the Comsol Multiphysics software.

For the calculation in the PEF mode, the following parameters were taken: the duty cycle, α, is equal to 0.5, and the PEF frequency, *f*, is equal to 0.5 Hz and 10 Hz. The authors [17] noted that under these conditions, an increase of the specific permselectivity of the membrane is observed. The frequency of 10 Hz was taken since, as follows from the work of Sistat et al. [16], the mass transfer rate in PEF mode increases with increasing frequency, and at *f* ≥ 10 Hz, this rate becomes nearly constant.

The model allows calculation of the ion concentration profiles and the electric current density in the membrane system. It is also possible to calculate the partial current densities of ions and their effective transport numbers, *T_k_*, in the membrane. *T_k_* is defined as a fraction of current transported by ion *k*:

(9) $$T_{k}=\frac{z_{k}J_{k}F}{i},$$

where $z_{k}J_{k}F=i_{k}$ is the partial current density of ion *k*.

## 4. Results and Discussions

All the calculations are carried out using the input parameters presented in Table 2. The output parameters are time-dependent concentrations profiles, total current density (when the voltage is the input parameter) or the voltage (if the current density is given) and the partial current densities, *i_k_* (or effective transport numbers, *T_k_*), computed in the membrane at a distance of 1 μm from the depleted solution/membrane interface (left-hand interface). The latter shows the rate of ion transport through the left-hand interface. The point located at 1 μm is chosen since, at a shorter distance, the errors in the calculated values of *i_k_* (*T_k_*) are relatively high due to very large concentration and potential gradients.

The diffusion coefficients of Na^+^ and Ca^2+^ in the membrane are determined from the electrical conductivity of a Neosepta CMX membrane [17]. The activity coefficients of the cations in the membrane are chosen from the following consideration. The activity coefficients in the membrane and solution are linked with the ion-exchange constant, *K_N_* (which is also called the Nikolsky’s constant [43,44]) as follows [45,46]:

(10) $$K_{N}={\left(\frac{{\bar{\gamma }}_{1}}{\gamma_{1}}\right)}^{\frac{1}{z_{1}}}{\left(\frac{\gamma_{2}}{{\bar{\gamma }}_{2}}\right)}^{\frac{1}{z_{2}}}.$$

*K_N_* enters the relation of local thermodynamic equilibrium, which holds at the solution/membrane interfaces:

(11) $${\bar{c}}_{2}{}^{1/z_{2}}/{\bar{c}}_{1}{}^{1/z_{1}}=K_{N}c_{2}{}^{1/z_{2}}/c_{1}{}^{1/z_{1}}.$$

The activity coefficients in the solution ($\gamma_{1}$ and $\gamma_{2}$) were taken equal to 1; the activity coefficients in the membrane (${\bar{\gamma }}_{1}$ and ${\bar{\gamma }}_{2}$) were found in a way that the equivalent fraction of Ca^2+^ in the membrane is about 10 times higher than that of Na^+^ (as experiment shows in the case of ion-exchangers with sulfonate fixed groups [45]), when their equivalent fractions in the equilibrium bathing solution are the same at *I* = 0 and when the concentrations of both ions in the solution were 0.02 eq/L, which approximately corresponds to the experimental conditions [17].

The limiting current density, *i*_lim_, of the system was calculated using Equation (12). This equation is applied for systems with ternary electrolyte (e.g., CaCl_2_ and NaCl), under the assumption that the membrane is impermeable to coion [47]:

(12) $$i_{\lim}^{}=\frac{F}{\delta }\sum_{k=1}^{2}\left(1-\frac{z_{k}}{z_{A}}\right)D_{k}z_{k}c_{k0}^{},$$

here index *k* is related to counterions (Na^+^ or Ca^2+^) and A to coion (Cl^−^); *δ* is the thickness of the depleted diffusion layer (*δ* = *δ^I^*), which was estimated from the hydrodynamic parameters of the experimental system [17]. Since the model does not take into account all the components of the experimental solution, in particular K^+^, which is the dominant cation [17], the calculated limiting current density *i*_lim_ = 6.52 mA/cm^2^ is lower than the experimental one (13.5 mA/cm^2^). However, in this paper, we are not aiming for quantitative agreement with the experiment. The goal is to understand whether taking into account electromigration and diffusion is sufficient to obtain a qualitatively correct picture of the effect of PEF on membrane permselectivity for a specific ion.

### 4.1. CC Mode

The case where constant voltage pulses alternate with pauses, during which the current density is zero (*i =* 0), is considered since it is often used in practice [14,16]. In this case, the results are compared at the same value of the time–average voltage, *U*_av_, which is linked with the voltage during the pulse, *U*_pulse_, as *U*_av_ = α*U*_pulse_.

**Figure 2:**
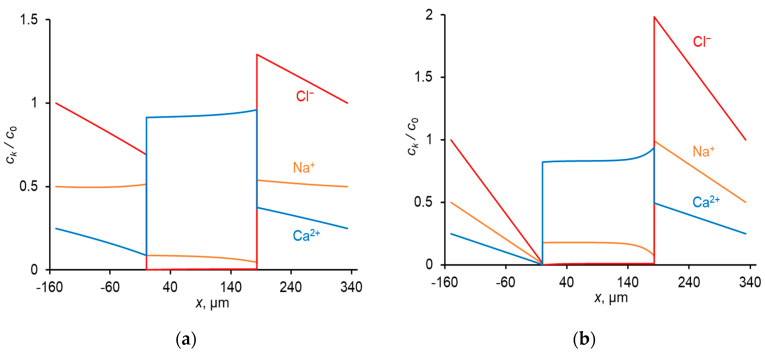
Concentration profiles of ions in CC mode at: *U*_av_ = 111 mV (*i* = 0.3 *i*_lim_) (**a**); *U*_av_ = 476 mV (*i* = *i*_lim_) (**b**). The concentrations are normalized by *c*_0_; *c*_0_ = $c_{Cl}^{0}$ in the diffusion layers ($c_{Cl}^{0}$ is the concentration of Cl^−^ ions in the bulk solution), and *c*_0_ = *Q* in the membrane.

**Figure 3:**
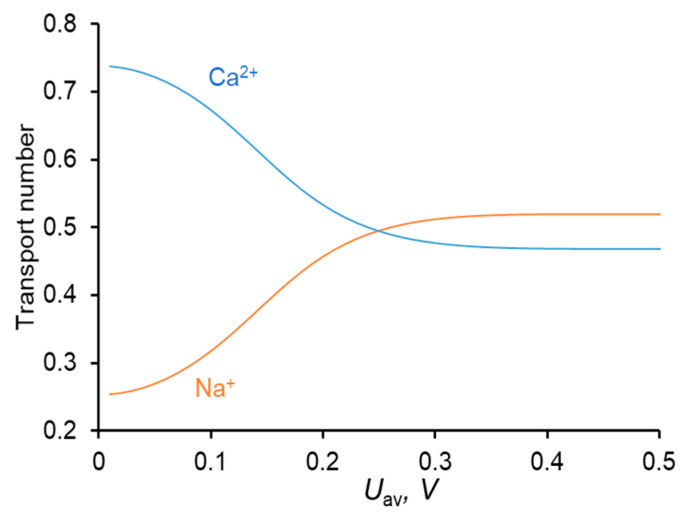
Dependence of the transport numbers of Ca^2+^ and Na^+^ ions in the membrane on the time–average voltage.

A relatively high concentration of Ca^2+^ in the membrane (due to great value of *K*_N_, Table 2) leads to a higher flux of this ion compared to the Na^+^ flux in conditions where the equivalent concentrations of both ions in the solution bulk are the same; thus, the membrane shows permselectivity for specific ions [48], the divalent Ca^2+^ cation in the considered case. However, with increasing current density, the permselectivity decreases, and it is nearly completely lost at *I = i*_lim_ [33,34,47]. This loss is due to a particular developing concentration polarization of the membrane. Since Ca^2+^ is the preferentially transferred ion over Na^+^, the applied electric current causes a faster decrease in the concentration of Ca^2+^ at the depleted solution/membrane interface than the decrease in the concentration of Na^+^. Moreover, the computation shows (and experimental data [49,50] confirm that) that at low voltages (e.g., at *U*_av_ = 111 mV in Figure 2a), the concentration of Na^+^ at the depleted interface can be even higher than its bulk value. Thus, under an applied current, the equilibrium at this interface shifts in favor of Na^+^, whose relative concentration in the depleted solution and in the membrane increases. As a result, the ratio between the fluxes of Ca^2+^ and Na^+^ (and between the transport numbers) changes in favor of Na^+^: preferential transfer of Ca^2+^ at low current densities (low voltages) is lost, the transfer rate of Na^+^ at *i* = *i*_lim_ becomes even greater than that of Ca^2+^ (Figure 3). At the limiting current density, the fluxes of the competing ions are controlled by the depleted diffusion layer and do not depend on the membrane properties Equation (9); the role of the membrane in terms of selective transfer is reduced to being a barrier for co-ions.

The shape of the *T_k_* vs. *U*_av_ dependence is in good agreement with the literature data [33,34].

### 4.2. PEF Mode (Current Pulses)

**Figure 4:**
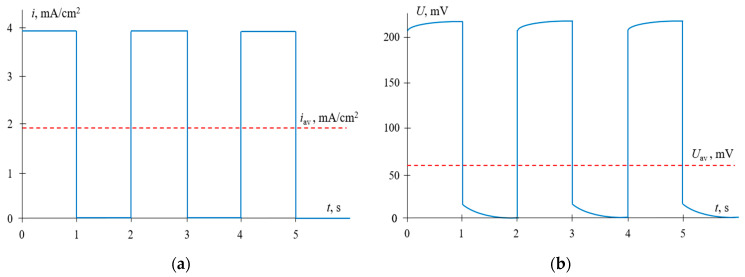
Time dependences of the current density (**a**) and voltage (**b**) for the pulsed electric field (PEF) mode, when current pulses are applied. *i*_av_ is the time–average current density; *U*_av_ is the average voltage. The results of calculations at *α* = 0.5, *f* = 0.5 Hz and *i*_CC_ = *i*_pulse_ = 0.6*i*_lim_.

Typical time dependences of the current density and voltage for the mode under consideration are shown in Figure 4.

**Figure 5:**
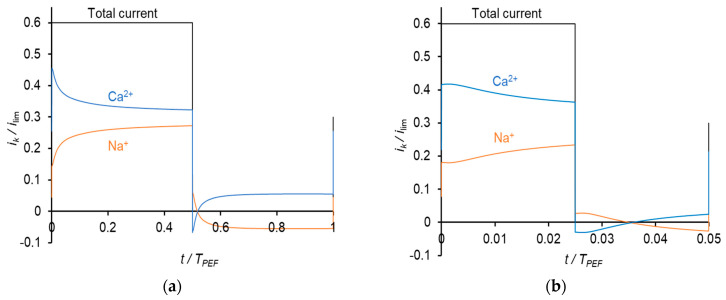
Dependences of partial current densities of Na^+^ and Ca^2+^ ions on time at the frequencies of PEF of 0.5 Hz (**a**) and 10 Hz (**b**) at *i*_av_ = 0.3 *i*_lim_.

The calculation shows that in the conditions of the experiment [17], when the same current density in CC mode and during the pulse is applied, *i*_CC_ = *i*_pulse_ = 0.6*i*_lim_, in CC mode, the T_Ca_:T_Na_ ratio is 0.52:0.48, while in PEF mode this ratio is essentially higher: 0.65:0.34; the parameters of the PEF mode are as follows: the duty cycle *α* = 0.5 and frequency *f* = 0.5 Hz. This theoretical result is in qualitative agreement with the experiment [17] described above (Section 2). The PEF effect is due to partial restoration of concentrations during a pause lapse. The restoration occurs near the surface; a little increase in concentration produces an important decrease in resistance. As a consequence, after a pause, the profiles are closer to those, which occur at a lower CC current density, which explains a higher permselectivity. An important feature is that the Na^+^ partial current density is negative during the pause lapse, while the Ca^2+^ partial current density remains positive (Figure 5a). This explains the increase in permselectivity attained in the PEF mode. However, when calculating the values of the transport numbers in the CC and PEF modes under the condition that the average over the period current density *i*_av_ in PEF mode is equal to the constant current density in CC mode, no difference was found between the transport numbers in CC and PEF modes. When we have taken *i*_av_ *= i*_CC_ = 0.3*i*_lim_ (in this case the current density during the pulse is *i*_pulse_ = 0.6*i*_lim_), *α* = 0.5 and *f* = 0.5 Hz, we find that the T_Ca_:T_Na_ ratio is the same in both modes and equal to 0.65:0.34. The same T_Ca_:T_Na_ ratio (0.65:0.34) is found at a higher frequency *f* = 10 Hz (*α* = 0.5), when *i*_pulse_ = 0.6*i*_lim_. The calculations show that the T_Ca_:T_Na_ ratio depends only on the *i*_av_ value and does not depend on the frequency and duty cycle; it does not matter if PEF or CC mode is used.

### 4.3. PEF Mode (Voltage Pulses)

**Figure 6:**
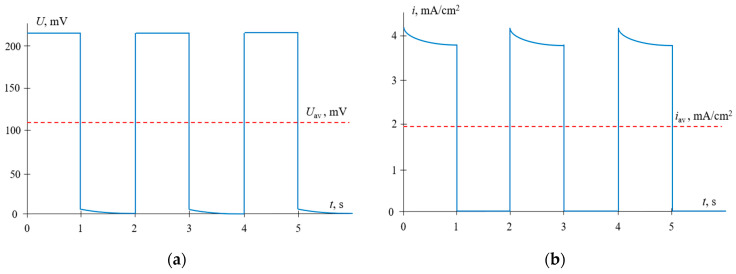
Time dependences of the voltage (**a**) and current density (**b**) for PEF mode, when voltage pulses are applied. The results of calculations at *α* = 0.5, *f* = 0.5 Hz and *U*_av_ = 111 mV (*i* = 0.3 *i*_lim_ in CC mode).

Typical time dependences of the voltage and current density for the PEF mode, where constant voltage pulses alternate with the pauses during which the current is zero, are shown in Figure 6.

**Figure 7:**
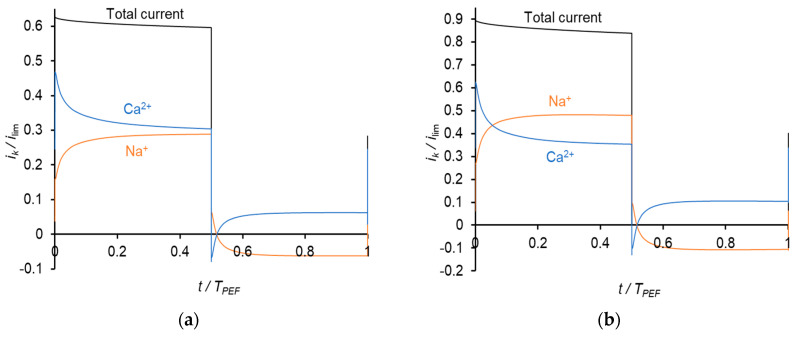

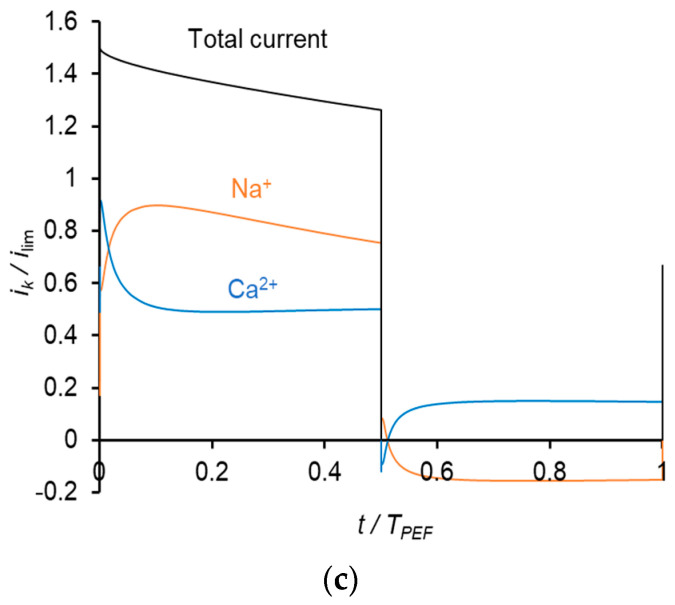
Time dependences of normalized partial current densities of Na^+^ and Ca^2+^ ions in PEF mode at a frequency of 0.5 Hz and different *U*_av_: *U*_av_ = 111 mV (*i* = 0.3 *i*_lim_ in CC mode) (**a**); *U*_av_ = 150 mV (*i* = 0.4 *i*_lim_ in CC mode) (**b**) and *U*_av_ = 238 mV (*i* = 0.65 *i*_lim_ in CC mode) (**c**). *T*_PEF_ is the period PEF.

The time dependences of the partial current densities of Na^+^ and Ca^2+^ ions at a frequency of 0.5 Hz are shown in Figure 7.

During a pulse lapse, the partial current density of Ca^2+^ gradually decreases, while that of Na^+^ increases. Qualitatively, this behavior is similar to that occurring in PEF mode, when constant current pulses are applied. Similar to above, the reason for the Ca^2+^ current diminution is a more rapid decrease in its concentration compared to that of Na^+^ in the depleted diffusion layer and membrane. During a pause lapse, the partial current density of Ca^2+^ increases, while that of Na^+^ decreases so that *i*_Na_ becomes negative. It should be noted that the total current density during pulse lapse is significantly higher than that at CC mode at the same average value of the voltage, *U*_av_.

**Figure 8:**
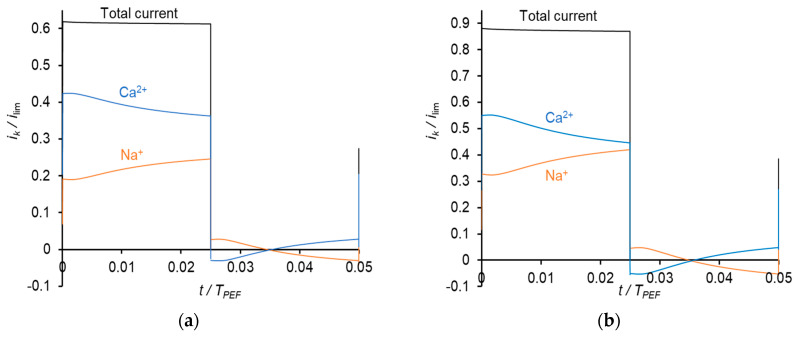

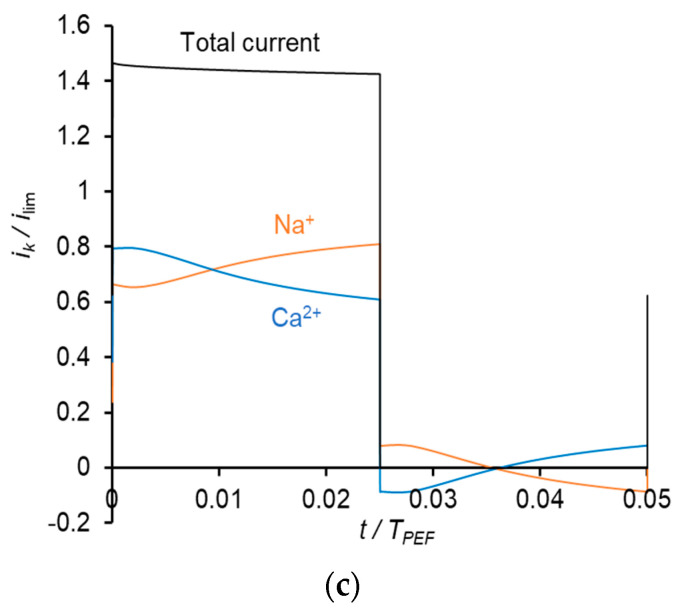
Dependences of normalized partial current densities of Na^+^ and Ca^2+^ ions on time in PEF mode at a frequency of 10 Hz and different *U*_av_: *U*_av_ = 111 mV (**a**); *U*_av_ = 150 mV (**b**) and *U*_av_ = 238 mV (**c**).

Similar results were obtained at the frequency of PEF of 10 Hz (Figure 8). However, due to a shorter duration of the pulse, the Na^+^ current density during a pulse lapse becomes greater than the current density of Ca^2+^ at a higher value of the average voltage (Figure 8c) than in the case of 0.5 Hz (Figure 7b).

The calculations show (Table 3) that the average current density, *i*_av_, in PEF mode is higher than that in CC mode at a given average voltage, *U*_av_; moreover, *i*_av_ increases with increasing frequency that agrees with the results of Sistat et al. [16]. This increase is explained by the fact that only the first moments (a few hundredths of a second) are profitable for obtaining a high current density (Figure 6b). With increasing duration of the pulse, the current density decreases rapidly due to increasing concentration polarization. However, when comparing the T_Ca_:T_Na_ ratios obtained in PEF mode and CC mode, it is found that this ratio is independent of how the PEF mode is applied, whether current or voltage pulses are applied, and what are the frequency and duty cycle. This ratio is a function of only one quantity, the average current density.

To summarize, note that our simulation results are consistent with the experiment of Lemay et al. [17] regarding the comparison of CC and PEF modes, provided that the current density during the pulse is the same as in the CC mode. In this case, experiment and calculation show that the membrane permselectivity for Ca^2+^ is improved in PEF mode. However, there is controversy about the effect of the frequency. The simulation shows that the membrane specific permselectivity in PEF mode does not depend on the frequency; only the average current density is important. However, the experiment indicates that under conditions that *i*_av_ is the same, the permselectivity for Ca^2+^ in the case of *f* = 0.5 Hz is greater than in the case of *f* = 5 Hz. Evidently, the mechanism of the effects observed in [17] is more complicated than the developed model suggests. It is possible that an important role in these phenomena is played by electroconvection. As mentioned in the introduction, at overlimiting average currents, electroconvection can increase the mass transfer rate by about 33% [25,26]. Electroconvective micro-vortices could essentially improve mixing the depleted solution near the membrane surface. The enhancement of convective mass transfer from the bulk to the membrane surface could shift the Ca^2+^:Na^+^ concentration ratio in favor of Ca^2+^. However, modeling competitive ion transport with taking into account electroconvection is not an easy task; it can be the subject of another publication.

## 5. Conclusions

A mathematical model based on the Nernst–Planck and Poisson equations is reported to describe competitive counterion transport through an ion-exchange membrane. CC mode and PEF mode are compared. Two regimes of PEF mode are studied: when constant current pulses or constant voltage pulses alternate with pauses during which the current is zero. It is shown that when the same current density is applied in CC mode and during the pulses in PEF mode, the permselectivity of the membrane for a specific counterion is higher in the case of PEF mode. This result is in agreement with the experimental data obtained by Lemay et al. [17] in the ED demineralization of sweet whey. However, when comparing the results of the simulation, provided that the current density in CC mode is the same as the average current density in PEF mode, it is found that effective transport numbers of two competing ions (*T*_1_ and *T*_2_) in PEF mode are equal to their values in CC mode. Moreover, the values of *T*_1_ and *T*_2_ do not depend on whether current or voltage is applied during the pulses; they are also independent of the frequency and duty cycle used in PEF mode. The last results do not agree with the experiment of Lemay et al. [17]. It was found in [17], that the membrane permselectivity for Ca^2+^ in the case of *f* = 0.5 Hz is greater than in the case of *f* = 5 Hz. Therefore, taking into account only electromigration and diffusion as the mechanisms of ion transport seems insufficient. It is possible that this discrepancy is due to electroconvection. Electroconvective micro-vortices could deliver a fresh solution from the solution bulk to the depleted membrane interface, which can maintain relatively high Ca^2+^:Na^+^ concentration and flux ratios at this interface.

As for the practical application of PEF mode in ED, this seems very promising. The main effect of PEF, proved by numerous authors, is the mitigation of the membrane scaling and fouling. In addition, the use of PEF mode leads to an increase in current efficiency and mass transfer and to a decrease in the membrane stack resistance. However, generally, since the current is zero during the relaxation pauses, the ED process in this mode takes more time than in the classical CC mode. Therefore, the optimization of PEF parameters (frequency, duty cycle) is needed. Some of these problems are discussed in the paper by Martí-Calatayud et al. [51].

The effect of PEF on the membrane permselectivity for specific ions is still not clear enough. The model presented in this paper provides an insight into the understanding of this effect. However, more efforts should be applied to explore the role of electroconvection and perhaps some other effects.

## Figures and Tables

**Table 1:** The total degree of desalination (DD) of sweet whey and the ratio of the DD by individual ions to the total DD, *θ_k_*.

Total DD	ED Mode	K^+^	Na^+^	Ca^2+^	Mg^2+^
30% *i/i*_lim_ ≈ 0.6	CC mode	1.3	0.57	0.67	0.57
	PEF mode, 1 s: 1 s	1.3	0.17	1.0	0.88
60% *i/i*_lim_ ≈ 1.5	CC mode	1.2	0.90	0.67	0.58
	PEF mode, 1 s: 1 s	1.2	0.61	0.87	0.83

**Table 2:** Input parameters used in the calculations.

Name	Value	Description
*c*_0_	0.02 mol/L	Bulk solution concentration
$\bar{Q}$	1.64 mol/L	Exchange capacity of the membrane
*D*_Na_	1.33 × 10^−9^ m^2^/s	Ion diffusion coefficients in the solution
*D*_Ca_	7.96 × 10^−10^ m^2^/s	
*D*_Cl_	2.04 × 10^−9^ m^2^/s	
${\bar{D}}_{Na}$	1.07 × 10^−10^ m^2^/s	Ion diffusion coefficients in the membrane
${\bar{D}}_{Ca}$	4.93 × 10^−12^ m^2^/s	
${\bar{D}}_{Cl}$	10^−11^ m^2^/s	
${\bar{\gamma }}_{Na}$	1	Ion activity coefficients in the membrane
${\bar{\gamma }}_{Ca}$	0.05	
${\bar{\gamma }}_{Cl}$	1	
*δ^I^*	150 μm	Depleted diffusion layer thickness
*δ^II^*	150 μm	Enriched diffusion layer thickness
*d*	180 μm	Membrane thickness
*K_N_*	4.5	Nikolsky’s constant

**Table 3:** Results of calculation of the average current density and transport numbers in the membrane in CC and PEF modes at different average voltages, *U*_av_, and different frequencies in Hz: *f* = 0 (CC mode), 0.5 and 10; α = 0.5.

*U*_av_, mV	*i*_av_/*i*_lim_			T_Ca_:T_Na_			T_Cl_		
	*f* = 0	*f* = 0.5	*f* = 10	*f* = 0	*f* = 0.5	*f* = 10	*f* = 0	*f* = 0.5	*f* = 10
111	0.3	0.303	0.307	0.646:0.345	0.629:0.363	0.639:0.352	0.0092	0.0085	0.0085
150	0.4	0.428	0.437	0.593:0.398	0.559:0.432	0.569:0.422	0.0093	0.0086	0.0086
238	0.65	0.678	0.720	0.501:0.490	0.485:0.506	0.482:0.508	0.0098	0.0092	0.0094

## Nomenclature

*Abbreviations*	
CC	Continuous current
DBL	Diffusion boundary layer
DD	Degree of desalination
ED	Electrodialysis
PEF	Pulsed electric field
PVC	Polyvinyl chloride
*Symbols*	
*c_k_*	Concentration of ion *k*
*c_k0_*	Concentration of ion *k* in the bulk solution
*d*	Membrane thickness
*D_k_*	Diffusion coefficient of ion *k* in the solution
${\bar{D}}_{k}$	Diffusion coefficient of ion *k* in the membrane
*E*	Electric field strength
*f*	Frequency
*F*	Faraday constant
*i*	Current density
*i_k_*	Partial current density of ion *k*
*i*_lim_	Limiting current density
*J_k_*	Flux density of ion *k*
*K_N_*	Nikolsky’s constant
$\bar{Q}$	Exchange capacity of the membrane
*R*	Universal gas constant
*t*	Time
*T*	Absolute temperature
*T_k_*	Effective transport number of ion *k* in membrane
*U*_av_	Average voltage
*U*_pulse_	Voltage, applied during the pulse
*z_k_*	Charge number of ion *k*
*z_m_*	Charge of fixed ions in the membrane
*Greek symbols*	
α	Duty cycle
$\gamma_{k}$	Activity coefficient of ion *k* in the solution
${\bar{\gamma }}_{k}$	Activity coefficient of ion *k* in the membrane
$\delta^{I}$,$\delta^{II}$	Thickness of the depleted and enriches diffusion boundary layers, respectively
ε	Solution relative permittivity
$\varepsilon_{0}$	Vacuum permittivity
$\theta_{k}$	Ratio of the degree of desalination by ion *k* to the total degree of desalination
φ	Electric potential

## References

[1] Strathmann H. (2010). Electrodialysis, a mature technology with a multitude of new applications. Desalination.

[2] Ran J., Wu L., He Y., Yang Z., Wang Y., Jiang C., Ge L., Bakangura E., Xu T. (2017). Ion exchange membranes: New developments and applications. J. Memb. Sci..

[3] Campione A., Gurreri L., Ciofalo M., Micale G., Tamburini A., Cipollina A. (2018). Electrodialysis for water desalination: A critical assessment of recent developments on process fundamentals, models and applications. Desalination.

[4] Apel P.Y., Bobreshova O.V., Volkov A.V., Volkov V.V., Nikonenko V.V., Stenina I.A., Filippov A.N., Yampolskii Y.P., Yaroslavtsev A.B. (2019). Prospects of Membrane Science Development. Membr. Membr. Technol..

[5] Mishchuk N.A., Koopal L.K., Gonzalez-Caballero F. (2001). Intensification of electrodialysis by applying a non-stationary electric field. Colloids Surfaces A Physicochem. Eng. Asp..

[6] Uzdenova A., Urtenov M. (2020). Potentiodynamic and galvanodynamic regimes of mass transfer in flow-through electrodialysis membrane systems: Numerical simulation of electroconvection and current-voltage curve. Membranes.

[7] Mikhaylin S., Nikonenko V., Pourcelly G., Bazinet L. (2014). Intensification of demineralization process and decrease in scaling by application of pulsed electric field with short pulse/pause conditions. J. Memb. Sci..

[8] Malek P., Ortiz J.M., Richards B.S., Schäfer A.I. (2013). Electrodialytic removal of NaCl from water: Impacts of using pulsed electric potential on ion transport and water dissociation phenomena. J. Memb. Sci..

[9] Zabolotskii V.I., Shel’deshov N.V., Gnusin N.P. (1988). Dissociation of Water Molecules in Systems with Ion-exchange Membranes. Russ. Chem. Rev..

[10] Simons R. (1979). Strong electric field effects on proton transfer between membrane-bound amines and water. Nature.

[11] Andreeva M.A., Gil V.V., Pismenskaya N.D., Dammak L., Kononenko N.A., Larchet C., Grande D., Nikonenko V.V. (2018). Mitigation of membrane scaling in electrodialysis by electroconvection enhancement, pH adjustment and pulsed electric field application. J. Memb. Sci..

[12] Asraf-Snir M., Gilron J., Oren Y. (2016). Gypsum scaling of anion exchange membranes in electrodialysis. J. Memb. Sci..

[13] Mikhaylin S., Bazinet L. (2016). Fouling on ion-exchange membranes: Classification, characterization and strategies of prevention and control. Adv. Colloid Interface Sci..

[14] Mishchuk N.A., Verbich S.V., Gonzales-Caballero F. (2001). Concentration Polarization and Specific Selectivity of Membranes in Pulse Mode. Colloid J..

[15] Dufton G., Mikhaylin S., Gaaloul S., Bazinet L. (2020). Systematic study of the impact of pulsed electric field parameters (Pulse/pause duration and frequency) on ED performances during acid whey treatment. Membranes.

[16] Sistat P., Huguet P., Ruiz B., Pourcelly G., Mareev S.A., Nikonenko V.V. (2015). Effect of pulsed electric field on electrodialysis of a NaCl solution in sub-limiting current regime. Electrochim. Acta.

[17] Lemay N., Mikhaylin S., Mareev S., Pismenskaya N., Nikonenko V., Bazinet L. (2020). How demineralization duration by electrodialysis under high frequency pulsed electric field can be the same as in continuous current condition and that for better performances?. J. Memb. Sci..

[18] Mikhaylin S., Nikonenko V., Pismenskaya N., Pourcelly G., Choi S., Kwon H.J., Han J., Bazinet L. (2016). How physico-chemical and surface properties of cation-exchange membrane affect membrane scaling and electroconvective vortices: Influence on performance of electrodialysis with pulsed electric field. Desalination.

[19] Nichka V.S., Geoffroy T.R., Nikonenko V., Bazinet L. (2020). Impacts of flow rate and pulsed electric field current mode on protein fouling formation during bipolar membrane electroacidification of skim milk. Membranes.

[20] Gao Q., Li Z., Lei C., Fu R., Wang W., Li Q., Liu Z. (2020). Application of pulsed electric field in antifouling treatment of sodium gluconate mother liquor by electrodialysis. Materials.

[21] Dufton G., Mikhaylin S., Gaaloul S., Bazinet L. (2019). Positive impact of pulsed electric field on lactic acid removal, demineralization and membrane scaling during acid whey electrodialysis. Int. J. Mol. Sci..

[22] Moya A.A., Moleón J.A. (2011). Application of the network simulation method to study the electrical response of ion-exchange membrane systems to pulsed electric fields. Desalination.

[23] Uzdenova A.M., Kovalenko A.V., Urtenov M.K., Nikonenko V.V. (2015). Effect of electroconvection during pulsed electric field electrodialysis. Numerical experiments. Electrochem. Commun..

[24] Rubinstein I., Zaltzman B., Kedem O. (1997). Electric fields in and around ion-exchange membranes. J. Memb. Sci..

[25] Butylskii D., Moroz I., Tsygurina K., Mareev S. (2020). Effect of surface inhomogeneity of ion-exchange membranes on the mass transfer efficiency in pulsed electric field modes. Membranes.

[26] Zyryanova S.V., Butyl’skii D.Y., Mareev S.A., Pis’menskaya N.D., Nikonenko V.V., Pourcelly G. (2018). Effect of Parameters of Pulsed Electric Field on Average Current Density through Nafion 438 Membrane in Electrodialysis Cell. Russ. J. Electrochem..

[27] Lemay N., Mikhaylin S., Bazinet L. (2019). Voltage spike and electroconvective vortices generation during electrodialysis under pulsed electric field: Impact on demineralization process efficiency and energy consumption. Innov. Food Sci. Emerg. Technol..

[28] Cifuentes-Araya N., Pourcelly G., Bazinet L. (2011). Impact of pulsed electric field on electrodialysis process performance and membrane fouling during consecutive demineralization of a model salt solution containing a high magnesium/calcium ratio. J. Colloid Interface Sci..

[29] Lee H.J., Moon S.H., Tsai S.P. (2002). Effects of pulsed electric fields on membrane fouling in electrodialysis of NaC1 solution containing humate. Sep. Purif. Technol..

[30] Cifuentes-Araya N., Astudillo-Castro C., Bazinet L. (2014). Mechanisms of mineral membrane fouling growth modulated by pulsed modes of current during electrodialysis: Evidences of water splitting implications in the appearance of the amorphous phases of magnesium hydroxide and calcium carbonate. J. Colloid Interface Sci..

[31] Park H.B., Kamcev J., Robeson L.M., Elimelech M., Freeman B.D. (2017). Maximizing the right stuff: The trade-off between membrane permeability and selectivity. Science.

[32] Wang P., Wang M., Liu F., Ding S., Wang X., Du G., Liu J., Apel P., Kluth P., Trautmann C. (2018). Ultrafast ion sieving using nanoporous polymeric membranes. Nat. Commun..

[33] Martí-Calatayud M.C., García-Gabaldón M., Pérez-Herranz V. (2012). Study of the effects of the applied current regime and the concentration of chromic acid on the transport of Ni^2+^ ions through Nafion 117 membranes. J. Memb. Sci..

[34] Golubenko D.V., Karavanova Y.A., Melnikov S.S., Achoh A.R., Pourcelly G., Yaroslavtsev A.B. (2018). An approach to increase the permselectivity and mono-valent ion selectivity of cation-exchange membranes by introduction of amorphous zirconium phosphate nanoparticles. J. Memb. Sci..

[35] Zabolotsky V.I., Manzanares J.A., Nikonenko V.V., Lebedev K.A., Lovtsov E.G. (2002). Space charge effect on competitive ion transport through ion-exchange membranes. Desalination.

[36] Mizutani Y., Yamane R., Ihara H., Motomura H. (1963). Studies of Ion Exchange Membranes. XVI. The Preparation of Ion Exchange Membranes by the “Paste Method.”. Bull. Chem. Soc. Jpn..

[37] Doi S., Yasukawa M., Kakihana Y., Higa M. (2019). Alkali attack on anion exchange membranes with PVC backing and binder: Effect on performance and correlation between them. J. Memb. Sci..

[38] Hori Y., Nakatani T., Mizutani Y. (1986). Morphology of ion exchange membranes. Microscopy.

[39] Pismenskaya N.D., Nikonenko V.V., Melnik N.A., Shevtsova K.A., Belova E.I., Pourcelly G., Cot D., Dammak L., Larchet C. (2012). Evolution with time of hydrophobicity and microrelief of a cation-exchange membrane surface and its impact on overlimiting mass transfer. J. Phys. Chem. B.

[40] Sarapulova V.V., Titorova V.D., Nikonenko V.V., Pismenskaya N.D. (2019). Transport Characteristics of Homogeneous and Heterogeneous Ion-Exchange Membranes in Sodium Chloride, Calcium Chloride, and Sodium Sulfate Solutions. Membr. Membr. Technol..

[41] Moya A.A. (2014). Electrochemical impedance of ion-exchange membranes in ternary solutions with two counterions. J. Phys. Chem. C.

[42] Uzdenova A., Kovalenko A., Urtenov M., Nikonenko V. (2018). 1D mathematical modelling of non-stationary ion transfer in the diffusion layer adjacent to an ion-exchange membrane in galvanostatic mode. Membranes.

[43] Scholz F. (2011). Nikolsky’s ion exchange theory versus Baucke’s dissociation mechanism of the glass electrode. J. Solid State Electrochem..

[44] Sillanpää M., Shestakova M. (2017). Emerging and Combined Electrochemical Methods.

[45] Helfferich F.G. (1995). Ion Exchange.

[46] Titorova V.D., Mareev S.A., Gorobchenko A.D., Gil V.V., Nikonenko V.V., Sabbatovskii K.G., Pismenskaya N.D. (2021). Effect of current-induced coion transfer on the shape of chronopotentiograms of cation-exchange membranes. J. Memb. Sci..

[47] Nikonenko V.V., Zabolotskii V.I., Gnusin N.P. (1980). Effect of stationary external electric fields on ion-exchange membrane selectivity. Sov. Electrochem..

[48] Sata T. (1994). Studies on ion exchange membranes with permselectivity for specific ions in electrodialysis. J. Memb. Sci..

[49] Shaposhnik V.A., Vasil’eva V.I., Praslov D.B. (1995). Concentration fields of solutions under electrodialysis with ion-exchange membranes. J. Memb. Sci..

[50] Vasil’eva V.I., Shaposhnik V.A., Grigorchuk O.V., Malykhin M.D. (2002). Electrodialysis kinetics by laser interferometry. Russ. J. Electrochem..

[51] Martí-Calatayud M.C., Sancho-Cirer Poczatek M., Pérez-Herranz V. (2021). Trade-Off between Operating Time and Energy Consumption in Pulsed Electric Field Electrodialysis: A Comprehensive Simulation Study. Membranes.

